# Antibody-Mediated Rejection in Kidney Transplantation: A Retrospective Study on the Impact of Donor-Specific Antibodies and on the Timing of Diagnosis

**DOI:** 10.7759/cureus.30296

**Published:** 2022-10-14

**Authors:** Pedro Reis Pereira, Bárbara Ribeiro, João Oliveira, Sofia Santos, Sofia Pedroso, Sandra Tafulo, Manuela Almeida, Leonídio Dias, La Salete Martins, Jorge Malheiro

**Affiliations:** 1 Nephrology, Centro Hospitalar de Trás-os-Montes e Alto Douro, Vila Real, PRT; 2 Nephrology, Dialysis, and Transplantation, Unit for Multidisciplinary Research in Biomedicine, Instituto de Ciências Biomédicas Abel Salazar (ICBAS), Porto, PRT; 3 Integrative and Translational Research, Laboratory for Integrative and Translational Research in Population Health, Porto, PRT; 4 Nephrology, Hospital de Braga, Braga, PRT; 5 Nephrology, Centro Hospitalar Universitário do Porto, Porto, PRT; 6 Blood and Transplantation Center of Porto, Portuguese Institute for Blood and Transplantation, Porto, PRT

**Keywords:** banff classification, kidney transplantation, transplantation outcomes, antibody-mediated rejection, donor-specific anti-hla antibody

## Abstract

Introduction

Limited information exists concerning the clinical significance of histologically confirmed antibody-mediated rejection (h-AMR) without detectable circulating donor-specific antibodies (DSA). In this study, we compared the outcomes of patients with h-AMR according to DSA status.

Methods

A total of 80 kidney transplant (KT) recipients who met the 2018 Banff criteria for h-AMR were included. Clinical and immunological characteristics were evaluated, and outcomes were compared according to DSA status after kidney biopsy (KB).

Results

There were 57 patients who had DSA-positive (+) h-AMR and 23 patients who had DSA-negative (-) h-AMR. Groups had similar baseline characteristics and time between KT and KB. Concerning histopathological diagnoses/Banff scores, DSA+ patients had higher interstitial fibrosis (ci) and tubular atrophy (ct) (ci+ct) scores and lower arterial hyalinosis (ah) scores compared to DSA- patients. Graft survival (GS) was similar for both groups (64% versus 44% at five years and 44% versus 34% at 10 years). Multivariate analysis revealed the time of KB (less than six months after KT or more than six months after KT) to be associated with GS. A stratified analysis was conducted, targeting DSA status according to the time of biopsy. For KB performed less than six months after KT, GS was higher for DSA+ patients at 10 years (66% versus 23%). For KB performed more than six months after KT, DSA- patients had higher GS at 10 years (58% versus 9%).

Conclusion

Both the timing of AMR diagnosis and DSA status had an impact on AMR outcomes. For patients diagnosed with AMR more than six months after transplantation, GS was worst for those in which circulating DSA were identified. Biopsy specimens from DSA- specimens had higher ct-ci and ah scores.

## Introduction

Antibody-mediated rejection (AMR) is the main cause of kidney graft dysfunction and graft failure after a kidney transplant (KT) [1]. Banff criteria for the diagnosis of AMR include allograft tissue injury, evidence of current or recent interaction of an antibody with the endothelium, and evidence of circulating antibodies [2]. However, in the setting of clinical suspicion of AMR, it is common that these criteria are not completely met because donor-specific antibodies (DSA) against human leukocyte antigen (HLA) are not detected in a significant proportion of patients [3-5].

It is becoming increasingly recognized that histological AMR (h-AMR) may be present if only the first two criteria are met, with or without detectable circulating DSA or complement split product 4d (C4d) staining [6,7]. Allograft histology of AMR shows endothelial activation, with glomerular basement membrane duplication, with or without C4d-positive staining, and/or microvascular inflammation [8,9]. Implications of DSA status in cases of proven h-AMR concern not only prognostic significance, in which previous studies have shown conflicting results [10-12], but also treatment options in DSA-negative (-) cases [13], as these patients are frequently excluded from clinical trials.

In this study, we compared the outcomes of patients with h-AMR according to DSA status.

## Materials and methods

Study population

We retrospectively reviewed the clinical and immunological characteristics of 80 kidney transplant (KT) recipients who met the 2018 Banff criteria for h-AMR [14] upon cause biopsy performed due to allograft function impairment. Patients were identified through our center’s renal pathology database between 2008 and 2018.

Patients were divided into two groups according to DSA status at the time of biopsy. Baseline demographic, anthropomorphic, analytical, and clinical data were collected. Renal allograft biopsies were re-evaluated, and lesions were scored according to the 2018 Banff classification. Transplant data and treatment approach were also analyzed. All patients were followed up until the end of June 2021, the date of death, the date of graft loss, or patients were lost during follow-up. The study protocol was reviewed and approved by the institutional ethical review and hospital administration boards in accordance with the recommendations of the Declaration of Helsinki and European Data Protection Regulations.

Detection and characterization of HLA antibodies

All patients were tested for DSA presence before transplant and at the time of h-AMR diagnosis by IgG and C1q single antigen bead (SAB) assays (LabScreen Single Antigen Beads®, One Lambda, Inc., Los Angeles, CA, USA). To account for a possible complement interference or prozone effect, all samples were treated with ethylenediaminetetraacetic acid. The mean fluorescence intensity (MFI) of each bead was measured using LABScan 100 flow analyzer (Luminex, Austin, TX, USA). A positive reaction threshold for IgG-SAB was considered an MFI value of ≥1,000. For C1q-SAB, antibodies were assigned as positive at 500 MFI or greater [15]. Donor and recipient were typed before transplant in loci HLA-A, HLA-B, and HLA-DR using polymerase chain reaction amplification with specific sequence primers (SSP) (Olerup SSP® low-resolution HLA typing kits, Stockholm, Sweden). Donor and recipient HLA-Cw, HLA-DQA1 and DQB1, and HLA-DP antigens were also typed for this study by SSP DNA typing when the recipient was sensitized against antigens from these loci. High-resolution typing was performed when necessary to establish whether a given anti-HLA antibody was donor-specific. For every individual DSA, the reported strength is based on the MFI of one single-antigen bead. In cases where more than one bead corresponding to the donor type was present within the panel, we recorded the one with the highest MFI level. In the case of more than one detectable DSA (IgG- or C1q-DSA) against different HLA antibodies, we defined DSA strength as the sum of all individual DSA MFI values [16].

Donor-specific antibodies detected in pretransplant and at the time of AMR diagnosis sera were compared. In 27 cases, one or more DSA identified at the time of biopsy were also present before transplantation; these patients were classified as having preformed DSA. Thirty patients had no DSA at the time of transplant and were classified as de novo DSA AMR cases.

Kidney graft histopathology

A single nephropathologist (SS), blinded to clinical information, evaluated all biopsies and categorized them using the 2018 Banff classification. As such, data on histopathological scores were recorded as follows: interstitial inflammation (i) and tubulitis (t) (i+t), endarteritis (v), glomerulitis (g), peritubular capillaritis (ptc), C4d deposition on peritubular capillaries, interstitial fibrosis (ci) and tubular atrophy (ct) (ci+ct), transplant glomerulopathy (cg), arterial hyalinosis (ah), and total inflammation (ti). Specimens were routinely stained with hematoxylin and eosin, periodic acid-Schiff, and methenamine silver (Jones) for light microscopic examination. Results on staining for complement split product 4d (C4d) were available by immunofluorescence in all cases [16].

AMR treatment

The decision on the treatment approach was based on patients’ clinical and graft histology characteristics and performed as previously described in our protocol for AMR [16]. Graft function, the presence of circulating DSA, biopsy positivity for C4d, the level of chronicity of biopsy findings, and patients’ comorbidities were individually considered. Most patients were treated with pulse steroids (500 mg of methylprednisolone for three days) plus antithymocyte globulin if the v score was 1 or greater. Some patients, mainly patients with evidence of circulating DSA, received also intravenous immunoglobulin therapy (IVIg) 2 g/kg (maximum: 140 g) divided into 2-4 doses associated with plasmapheresis (at least three sessions), with some of them being given additionally rituximab (four doses of 375 mg/m^2^). All cases received increased maintenance immunosuppression after AMR diagnosis.

Renal allograft function

The Chronic Kidney Disease Epidemiology Collaboration (CKD-EPI) equation was used to predict the estimated glomerular filtration rate (eGFR) before renal allograft biopsy. Delayed graft function (DGF) was defined as the need for at least one dialysis session in the first week after transplantation.

Statistical analysis

Continuous data are described as mean±standard deviation (SD) or median (interquartile range (IQR)), and categorical data are expressed as numbers and percentages. Categorical data were compared using Pearson’s chi-square test or Fisher’s exact test, and continuous variables were compared using Student’s t-test or Mann-Whitney U test, as appropriate.

AMR and graft survival (GS) curves were visualized using the Kaplan-Meier method. Comparisons between patient groups were performed using the log-rank test. In cases of death with a functioning graft, the time was censored at the time of death. Potential predictors of AMR and graft failure were explored using univariate and multivariate Cox proportional hazard models. In all multivariable models, independent predictors were identified using a backward elimination method, with a P value < 0.05 necessary for retention in the model.

A two-sided P value of <0.05 was considered statistically significant. Statistical calculations were performed using STATA/MP version 15.1 (StataCorp, College Station, TX, USA).

## Results

We identified 80 KT recipients who met the 2018 Banff criteria for h-AMR from 2008 to 2018. From these, 57 (71%) patients were identified as DSA-positive (+) and 23 patients were identified as DSA-negative (-).

Both groups had similar time between KT and KB and similar baseline characteristics, except for retransplantation, which was significantly higher in the DSA+ group at 30% (n=17) than in the DSA- group at only 4% (n=1) (Table 1). The median receptor age was similar for both groups, 40 (36-48) years for the DSA- group and 43 (34-50) years for the DSA+ group. The average HLA-A, HLA-B, and HLA-DR mismatch (MM) were 3.78±1.17 for DSA- recipients and 3.72±1.33 for DSA+ recipients, and desensitization protocols before transplant (including plasmapheresis, rituximab, and intravenous immunoglobulin therapy (IVIG)) were used in 12% (n=7) of the patients in the DSA+ group and no patient in the DSA- group.

**Table 1:** Clinical and immunological characteristics of patient groups according to DSA status. AMR: antibody-mediated rejection; DSA: donor-specific antibodies; IQR: interquartile range; ABDR: HLA-A, HLA-B, and HLA-DR; MM: mismatch; ATG: antithymocyte globulin; IVIG: intravenous immunoglobulin; PP: plasmapheresis; Rtx: rituximab; KT: kidney transplant; Tac: tacrolimus; IS: immunosuppression; eGFR: estimated glomerular filtration rate; MFI: mean fluorescence intensity; PRA: panel reactive antibody; SD: standard deviation

	Total (n=80)	AMR DSA- (n=23)	AMR DSA+ (n=57)	P value
At transplant				
Recipient age, median (IQR)	42 (34-50)	40 (36-48)	43 (34-50)	0.722
Female recipient, n (%)	33 (41)	7 (30)	26 (46)	0.212
Female donor, n (%)	26 (33)	6 (26)	20 (35)	0.437
Donor age, median (IQR)	46 (37-56)	45 (38-57)	46 (36-56)	0.928
Living donor, n (%)	15 (19)	2 (9)	13 (23)	0.209
Retransplant, n (%)	18 (23)	1 (4)	17 (30)	0.016
Cytotoxic PRA > 5%, n (%)	11 (14)	1 (4)	10 (18)	0.163
ABDR MM, mean±SD	3.74±1.28	3.78±1.17	3.72±1.33	0.939
ATG induction, n (%)	35 (44)	8 (35)	27 (47)	0.304
Desensitization (IVIG+PP±Rtx), n (%)	7 (9)	0	7 (12)	0.184
At biopsy				
Years from transplant, median (IQR)	1.4 (0.1-7)	2.3 (0.3-7.2)	1.2 (0.1-7)	0.347
Biopsy < 6 months after KT, n (%)	33 (41)	8 (35)	25 (44)	0.455
Tac-based IS, n (%)	60 (75)	19 (83)	41 (72)	0.318
Previous rejection, n (%)	15 (19)	6 (26)	9 (16)	0.286
eGFR (mL/minute), median (IQR)	25.4 (18.7-39.5)	32.3 (20.4-47.1)	23.6 (16.5-38.5)	0.028
Proteinuria (g/g), median (IQR)	0.9 (0.3-1.5)	0.8 (0.3-1.6)	0.9 (0.3-1.4)	0.861
Treatment of rejection, n (%) (steroids)	64 (80)	14 (61)	50 (88)	0.007
Treatment of rejection, n (%) (ATG)	35 (44)	6 (26)	29 (51)	0.043
Treatment of rejection, n (%) (IVIG)	37 (46)	2 (9)	35 (61)	<0.001
Treatment of rejection, n (%) (PP)	27 (34)	2 (9)	27 (47)	<0.001
Treatment of rejection, n (%) (rituximab)	23 (29)	2 (9)	23 (40)	<0.001
Treatment of rejection, n (%) (IVIG±PP±Rtx)	37 (46)	2 (9)	35 (61)	<0.001
De novo DSA, n (%)			30 (53)	
DSA C1q+, n (%)			25 (44)	
DSA class, n (%)				
I			12 (21)	
II			26 (46)	
I+II			19 (33)	
Peak DSA MFI, median (IQR)			11,670 (6,021-18,816)	
Years of follow-up after biopsy, median (IQR)	3.9 (1.4-7.6)	5.7 (1.8-7.3)	2.9 (1.1-7.8)	0.531

Median years from transplant at biopsy was 2.3 (0.3-7.2) in the DSA- group and 1.2 (0.1-7) in the DSA+ group. The percentage of biopsies performed less than six months after KT was 35% (n=8) for DSA- patients and 44% (n=25) for DSA+ patients. The rate of previous rejections was 26% (n=6) for the DSA- group and 16% (n=16) for the DSA+ group. The median eGFR (mL/minute/1.73 m^2^) at the time of biopsy was 32.3 (20.4-47.1) for DSA- patients and 23.6 (16.5-38.5) for DSA+ patients, and the median proteinuria (g/g) at the time of biopsy was 0.8 (0.3-1.6) for DSA- patients and 0.9 (0.3-1.4) for DSA+ patients.

Maintenance immunosuppression regimen was tacrolimus-based for 83% (n=19) of DSA- patients and 72% (n=41) of DSA+ patients. Treatment of rejection included steroids in 61% (n=14) of DSA- patients and 88% (n=50) of DSA+ patients, antithymocyte globulin (ATG) in 26% (n=6) of DSA- patients and 51% (n=29) of DSA+ patients, IVIG in 9% (n=2) of DSA- patients and 61% (n=35) of DSA+ patients, plasmapheresis in 9% (n=2) of DSA- patients and 47% (n=27) of DSA+ patients, and rituximab in 9% (n=2) of DSA- patients and 40% (n=23) of DSA+ patients. Simultaneous treatment with IVIG, plasmapheresis, and rituximab for rejection was used in 9% (n=2) of DSA- patients and 61% (n=35) of DSA+ patients.

In DSA+ patients, the rate of de novo DSA was 53% (n=30), and the rate of C1q positivity was 44% (n=25). Concerning DSA classes, DSA+ patients had class I DSA in 21% (n=12) of cases, class II DSA in 46% (n=26) of cases, and class I and II DSA in 33% (n=19) of cases. The median peak DSA mean fluorescence intensity (MFI) was 11,670 (6,021-18,816).

The median follow-up time after KB was 5.7 (1.8-7.3) years for the DSA- group and 2.9 (1.1-7.8) years for the DSA+ group.

Regarding KB findings (Table 2), using the 2018 Banff criteria, antibody-mediated changes were similar between groups, with active AMR being present in 39% (n=9) of DSA- recipients and 46% (n=26) of DSA+ recipients and chronic active AMR being found in 61% (n=14) of DSA- patients and 54% (n=31) of DSA+ patients. Concomitant T-cell-mediated rejection (TCMR) was found in 30% (n=7) of DSA- KB and 19% (n=11) of DSA+ KB, which was also statistically similar for both groups. Positivity for C4d on biopsy tissue was only found in DSA+ patients at 49% (n=28). The mean ct+ci score was significantly higher in DSA- patients, as well as the ah score.

**Table 2:** Histopathological diagnoses/2018 Banff scores. AMR: antibody-mediated rejection; DSA: donor-specific antibodies; aAMR: active antibody-mediated rejection; caAMR: chronic active antibody-mediated rejection; TCMR: T-cell-mediated rejection; SEM: standard error of the mean

	Total (n=80)	AMR DSA- (n=23)	AMR DSA+ (n=57)	P value
Antibody-mediated changes, n (%)				0.597
aAMR	35 (46)	9 (39)	26 (46)	
caAMR	45(56)	14 (61)	31 (54)	
Concomitant TCMR, n (%)	18 (23)	7 (30)	11 (19)	0.280
Grade 1, n (%)	2 (3)	2 (9)	0	0.080
Grade 2, n (%)	16 (20)	5 (22)	11 (19)	0.805
AMR C4d+, n (%)	28 (35)	0	28 (49)	<0.001
t+i score, mean (SEM)	1.89 (0.13)	2.09 (0.31)	1.81 (0.14)	0.524
v score, mean (SEM)	0.26 (0.06)	0.30 (0.13)	0.25 (0.07)	0.766
g+ptc score, mean (SEM)	3.06 (0.15)	2.70 (0.28)	3.21 (0.18)	0.156
C4d score, mean (SEM)	1.08 (0.14)	0.13 (0.07)	1.46 (0.18)	<0.001
ti score, mean (SEM)	1.74 (0.10)	1.83 (0.18)	1.70 (0.12)	0.656
ct+ci score, mean (SEM)	2.05 (0.20)	2.61 (0.35)	1.82 (0.23)	0.045
cg score, mean (SEM)	1.20 (0.13)	1.39 (0.26)	1.12 (0.16)	0.390
ah score, mean (SEM)	0.76 (0.12)	1.17 (0.25)	0.60 (0.12)	0.029

**Figure 1:**
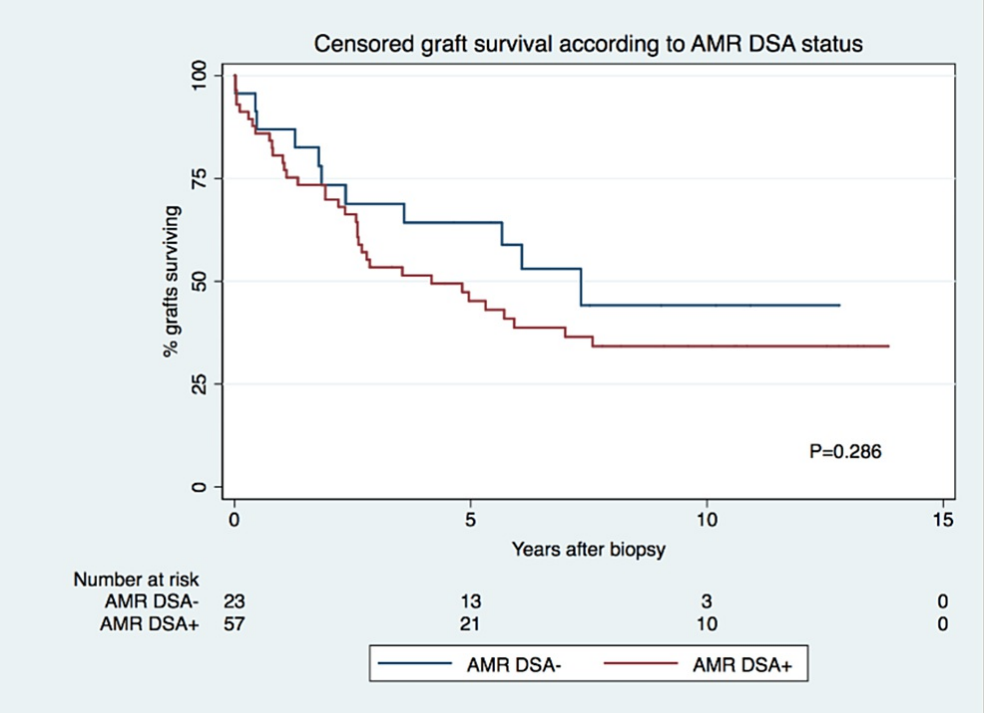
Survival according to DSA status. ABM: antibody-mediated rejection; DSA: donor-specific antibodies

**Figure 2:**
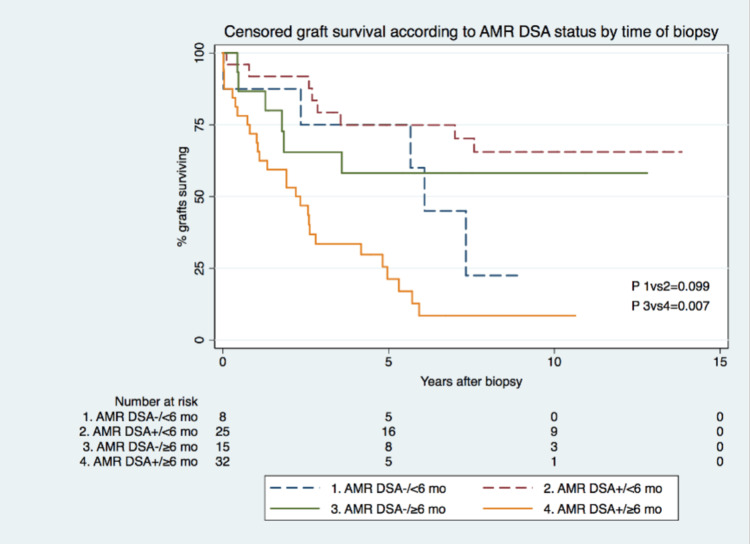
Survival according to DSA status and time frame of kidney biopsy. ABM: antibody-mediated rejection; DSA: donor-specific antibodies; mo: months

Censored GS was similar for both groups, at 64% versus 44% at five years and 44% versus 34% at 10 years, for the DSA- group and the DSA+, respectively (Figure 1). Multivariate analysis revealed that the time of KB had a prognostic value, with KB performed more than six months after KT associating with graft failure (hazard ratio (HR): 3.661; P<0.001) as opposed to KB performed less than six months after KT, regardless of DSA status (Table 3). Given the strong association between the time of biopsy (more than six months or less than six months after KT) and the known distinct nature of humoral injury within each of those time frames, a stratified analysis was conducted targeting DSA status according to the time of KB. For KB performed less than six months after KT, GS was the same for both groups at five years (75%) and was significantly higher for DSA+ patients at 10 years (66% versus 23%). For KB done more than six months after KT, GS was significantly higher for DSA- patients at five years (58% versus 21%) and was also significantly higher for DSA- patients at 10 years (58% versus 9%) (Figure 2). In biopsies performed more than six months after KT, positive DSA status was associated with a significantly higher risk of graft failure at 10 years (HR: 3.741; P<0.05), while for biopsies performed less than six months after KT, positive DSA status was associated with lower risk of graft failure (HR: 0.225; P<0.05). When comparing KB of subgroups in stratified analysis, for biopsies performed less than six months after KT, the rate of concomitant TCMR was significantly higher in DSA- cases at 75% (n=6) versus 24% (n=6) in DSA+ cases. KB characteristics on stratified analysis are presented in Table 4.

**Table 3:** Comparison of clinical and immunological characteristics between AMR DSA status stratified by time of biopsy. AMR: antibody-mediated rejection; DSA: donor-specific antibodies; Bx: biopsy; mo: months; eGFR: estimated glomerular filtration rate; IQR: interquartile range; ATG: antithymocyte globulin; IVIG: intravenous immunoglobulin; PP: plasmapheresis; Rtx: rituximab; MFI: mean fluorescence intensity; aAMR: active antibody-mediated rejection; caAMR: chronic active antibody-mediated rejection; TCMR: T-cell-mediated rejection; SEM: standard error of the mean

	AMR DSA- Bx < 6 mo (n=8) (1)	AMR DSA+ Bx < 6 mo (n=25) (2)	P value (1 versus 2)	AMR DSA- Bx ≥ 6 mo (n=25) (3)	AMR DSA+ Bx ≥ 6 mo (n=32) (4)	P value (3 versus 4)
Clinical data						
eGFR (mL/minute), median (IQR)	36.1 (20.3-48.6)	23.7 (15.8-38.5)	0.093	31.6 (21.6-41)	22.9 (18-38)	0.132
Proteinuria (g/g), median (IQR)	0.5 (0.2-0.7)	0.5 (0.3-1.2)	0.231	1.2 (0.4-3.1)	1 (0.4-2.1)	0.444
Treatment of rejection, n (%) (steroids)	8 (100)	25 (100)	1	6 (86)	15 (79)	1
Treatment of rejection, n (%) (ATG)	6 (75)	24 (96)	0.139	0	5 (16)	0.162
Treatment of rejection, n (%) (IVIG±PP±Rtx)	1 (13)	22 (88)	<0.001	1 (7)	13 (41)	0.020
DSA						
De novo DSA, n (%)		0			30 (94)	
DSA C1q+, n (%)		8 (32)			17 (53)	
DSA class, n (%)						
I		7 (28)			5 (16)	
II		7 (28)			19 (59)	
I+II		11 (44)			8 (25)	
Peak DSA MFI, median (IQR)		14,419 (6,000-22,836)			10,411 (6,092-17,021)	
Histological diagnoses						
Antibody-mediated changes, n (%)			1			1
aAMR	7 (88)	21 (84)		2 (13)	5 (16)	
caAMR	1 (13)	4 (16)		13 (87)	27 (84)	
Concomitant TCMR, n (%)	6 (75)	6 (24)	0.015	1 (7)	5 (16)	0.648
Grade 1, n (%)	1 (13)	0	0.242	1 (7)	0	0.319
Grade 2, n (%)	5 (63)	6 (24)	0.044	0	5 (16)	0.162
AMR C4d+, n (%)	0	18 (72)	<0.001	0	10 (31)	0.019
Banff scores						
t+i score, mean (SEM)	2.87 (0.61)	1.72 (0.20)	0.055	1.67 (0.30)	1.88 (0.20)	0.525
v score, mean (SEM)	0.88 (0.30)	0.32 (0.13)	0.046	0 (0)	0.19 (0.08)	0.110
g+ptc score, mean (SEM)	3.00 (0.63)	3.08 (0.29)	0.746	2.53 (0.27)	3.31 (0.24)	0.109
C4d score, mean (SEM)	0.13 (0.13)	2.16 (0.22)	<0.001	0.13 (0.09)	0.91 (0.22)	0.037
ti score, mean (SEM)	2.00 (0.33)	1.32 (0.18)	0.098	1.73 (0.23)	2 (0.13)	0.295
ct+ci score, mean (SEM)	1.75 (0.45)	0.72 (0.22)	0.033	3.01 (0.44)	2.69 (0.30)	0.439
cg score, mean (SEM)	0.25 (0.25)	0.28 (0.14)	0.840	2.00 (0.28)	1.78 (0.19)	0.490
ah score, mean (SEM)	0.63 (0.26)	0.12 (0.07)	0.020	1.47 (0.34)	0.97 (0.19)	0.217

**Table 4:** Independent predictors of graft failure by multivariable Cox regression. The multivariable model included all variables in Table 1 (except DSA data), with independent predictors being identified using a backward elimination method, with a P value < 0.05 necessary for retention in the model HR: hazard ratio; CI: confidence interval; eGFR: estimated glomerular filtration rate; KT: kidney transplant

	HR	95% CI	P value
Log-transformed eGFR at biopsy	0.298	0.149-0.593	0.001
Biopsy ≥ 6 (versus <6) months after KT	3.661	1.873-7.157	<0.001

## Discussion

AMR is the most important cause of kidney allograft failure and dysfunction after KT [1]. DSA are considered to have major importance in the pathogenesis of AMR but often remain undetectable in the serum of patients with proven h-AMR. In cases of proven h-AMR, DSA status may affect prognosis and influence therapeutic choices. In this study, we aimed to evaluate the impact of detecting circulating DSA on clinical outcomes.

In our retrospective study with KB performed upon cause, from all 80 patients identified as having h-AMR, 71% were DSA+ and 29% were DSA-. Our findings regarding the rate of patients with detectable DSA are consistent with the findings of previous studies, which reported rates ranging from 20% to 80% [9,17-19].

Censored GS was statistically similar between DSA+ and DSA- patients at 64% and 44% at five years and at 44% and 34% at 10 years, for the DSA- group and the DSA+ group, respectively (Figure 1). This finding is in line with previous studies with biopsies performed for cause that did not show DSA status to have a significant impact on GS [10,11,13]. However, in studies with protocol biopsies, findings have shown DSA- patients to have better GS [12,20].

Multivariate analysis revealed the time of KB (less than six months after KT or more than six months after KT) to be associated with GS (Table 3). A stratified analysis with DSA status and time of biopsy was performed. For KB performed less than six months after KT, GS was higher for DSA+ patients at 10 years (66% versus 23%), while for KB performed more than six months after KT, DSA- patients had higher GS at 10 years (58% versus 9%) (Figure 2). These findings challenge the view that the detection of DSA does not influence clinical outcomes. As we excluded patients with a diagnosis of h-AMR in the immediate post-transplantation period (less than six months after KT), we detected statistically significant differences in long-term outcomes between groups, with DSA+ patients showing worst GS, in line with studies with protocol biopsies. These differences were not detected in a crude analysis of GS according solely to DSA status, when not considering the timing of KB. In such cases, differences seem to be attenuated because if h-AMR is diagnosed in the immediate post-transplantation period (until six months after KT), DSA- patients would have the worst prognosis on the long term, which balances the negative long-term outcomes identified in the inverse analysis. Additionally, we found that for biopsies performed less than six months after KT, DSA- patients had a significantly higher proportion of concomitant TCMR (75% versus 24% in DSA+ patients). The hypothesis is that these patients actually have an important component of concomitant TCMR, DSA- status, and worst prognosis in the long term [21]. Furthermore, the treatment approach was different between groups (Table 4). In DSA+ patients, in cases of early h-AMR diagnosis, a significantly higher percentage of patients was treated with plasmapheresis, IVIG, and rituximab (88% versus 41% in DSA+ with late AMR diagnosis), and thus, the worst prognosis in DSA+ patients with KB more than six months after KT could be explained by a lower rate of anti-humoral treatment. However, the fact that the majority of the detected DSA in patients with late post-KT AMR diagnosis were de novo DSA and that a high rate of them (53%) were DSA C1q+, which are associated with the worst prognosis [22], may also have negatively impacted outcomes in this group. Eplet mismatch (MM) evaluation may be an opportunity to reduce de novo DSA formation, as recent studies have shown it to be more associated with eplet MM in HLA-DQ than in other loci [23].

Regarding the histomorphology characteristics of biopsy specimens (Table 2), both ct-ci and ah scores were higher in DSA- cases. These differences contrast with the findings from previous studies, which reported no differences in histomorphology between DSA+ and DSA- patients [10,11]. In a cohort study with 208 patients with proven h-AMR with protocol biopsies, although histological picture did not differ significantly between DSA+ and DSA- patients, DSA- patients were considered to have more transient h-AMR biopsy characteristics [12]. In another study with 224 protocol biopsies, transcriptional analysis of biopsy tissue revealed that h-AMR corresponded to a transcriptional signature, irrespective of DSA status [20]. In a recent study with 954 patients, although transplant glomerulopathy was shown to occur also in the absence of DSA, DSA- patients showed less concomitant interstitial inflammation, less glomerulitis, less C4d deposition in the peritubular capillaries, and better graft survival compared to patients with DSA [24]. More studies are needed to evaluate how DSA status impacts allograft histomorphology, and studies with protocol biopsies may help settle the contribution of the timing of diagnosis and the level of chronicity to outcomes.

The sample size is the most important limitation of the current study. The fact that the treatment approach was different between groups (as it is strongly influenced by the detection of DSA in circulation) should be underlined as another pitfall, as it is a bias that affects most studies in this area, and another proof of the impact of DSA status. Additionally, the fact that this was a retrospective study instead of a prospective study designed to access kidney transplant outcomes implies that the level of evidence is not as high as it would be if derived from a clinical trial. In opposition, our data concerns a single clinical center, with patients with similar demographic characteristics, submitted to kidney transplantation by the same team of professionals, with consistent standards of care, which increases the robustness of data retrieval and statistical analysis.

## Conclusions

Both the timing of AMR diagnosis and DSA status had an impact on AMR outcomes. For patients diagnosed with AMR more than six months after transplantation, GS was worst for those in which circulating DSA were identified. Biopsy specimens from DSA- specimens had higher ct-ci and ah scores.
